# Involvement of ascorbate oxidase in the regulation of the leaf development mediated by mepiquat chloride in cotton seedlings

**DOI:** 10.3389/fpls.2026.1899637

**Published:** 2026-07-31

**Authors:** Ye Tian, Xuanxuan Liu, Haiyan Guan, Leyue Li, Xiuxiu Jing, Xiaopin Zhu, Li Wang

**Affiliations:** 1 College of Life Sciences and Technology, North Henan Medical University, Xinxiang, China; 2 College of Life Sciences, Henan Normal University, Xinxiang, China

**Keywords:** ascorbate oxidase, ascorbic acid, cotton, leaf development, mepiquat chloride, transcriptome, virus-induced gene silencing

## Abstract

Mepiquat chloride (MC), a plant growth regulator, is extensively used in agricultural practices. However, the mechanism of action of MC is complex and remains largely unclear. In this study, the effects of MC on the leaf development of cotton seedlings and the underlying regulatory mechanism were investigated by integrating morphological, physiological, transcriptomic analysis and virus-induced gene silencing technology. The results showed that MC treatment caused a reduction in leaf area and an increase in chlorophyll content and photosynthetic efficiency. The ascorbic acid content initially increased and then declined after MC treatment. Transcriptomic data demonstrated that the expression of the ascorbate oxidase (AO) gene was initially upregulated and subsequently downregulated by MC. The top 20 KEGG pathways identified from the differentially expressed genes were mainly enriched in plant–pathogen interaction, the MAPK signaling pathway, ascorbate and aldarate metabolism, plant hormone signal transduction, amino acid metabolism, and secondary metabolism. Silencing *GhAO1* enhanced the ascorbic acid concentration, which was accompanied by increased plant height, internode length, and leaf area in cotton seedlings. Silencing *GhAO1* reduced the sensitivity of cotton seedlings to MC. This study reveals that MC may primarily serve as an abiotic stressor that regulates the stress response during the early stage of MC treatment and may further enhance chlorophyll content by promoting amino acid and secondary metabolism. AO plays an important role in MC-mediated growth regulation in cotton seedlings.

## 1 Introduction

Cotton (*Gossypium hirsutum* L.) is an important economic crop worldwide. In China, cultivated cotton accounts for more than 95% of the country’s fiber crop production and serves as the primary source of textile fibers (Wu et al., 2019). Therefore, optimizing cotton germplasm and increasing cotton fiber yield are of particular importance. However, due to its indeterminate growth habit, cotton is prone to excessive vegetative growth, which is characterized by the overdevelopment of branches and leaves, and the premature development of the canopy (Gu et al., 2014). Its self-shading limits the penetration of solar radiation to the lower leaves, thereby reducing photosynthetic efficiency and assimilate production. These factors make it difficult to achieve high-quality, high-yield cotton (Abbas et al., 2022).

Mepiquat chloride (MC) is a water-soluble compound widely used to regulate plant growth by interfering with the gibberellin (GA) signaling pathway (Luo and Tang, 2022). When applied during cotton growth, MC modulates the development of cotton by promoting root growth, enhancing seedling vigor, and reducing plant height (Luo and Tang, 2022). It effectively suppresses excessive vegetative growth, shaping a more ideal plant architecture and canopy structure (Gu et al., 2014). Consequently, cotton plants can more efficiently utilize environmental resources, such as light, temperature, water, and nutrients. This promotes early flowering and boll set, improves boll structure, accelerates maturity, and increases boll weight, ultimately enhancing both cotton yield and fiber quality (Tung et al., 2020; Wang et al., 2022b).

The effects of MC application on cotton growth vary depending on the developmental stage at which it is applied. Seed soaking with MC prior to sowing increases chlorophyll content in leaves, enhances light energy absorption and conversion, and elevates the photosynthetic rate (Wu et al., 2019). It also stimulates lateral root formation and elongation and increases root surface area and volume, thereby improving the root’s capacity for water and mineral uptake and promoting overall root development (Tung et al., 2018). Before and after the initial flowering period, MC primarily affects the outer internodes of fruiting branches below the N node (the main stem node at the time of MC application) and most internodes of fruiting branches above the N node (Zhao and Oosterhuis, 2000). Prior to the full flowering stage, MC reduces the total number of main stem nodes. After the squaring stage, MC influences the elongation of nearly all fruiting nodes on middle and lower fruiting branches, as well as some inner fruiting nodes on the upper branches (Cook and Kennedy, 2000). The effects of MC on plant growth are also species-dependent. Cotton is highly sensitive to MC, whereas maize exhibits relative insensitivity (Zhang et al., 2020). The *ent*-copalyl diphosphate synthase (CPS), a key enzyme in GA biosynthesis, is insensitive to MC in maize, whereas it is the primary site of MC action in cotton (Zhang et al., 2020). Even among cotton cultivars, responses to MC vary significantly (Wang et al., 2020b). Besides cotton, MC influences the growth of other species, such as soybean, sugarcane, pepper, tomato, and sunflower (Polat et al., 2017; Chen et al., 2021; Wang et al., 2022a; Geng et al., 2025; Wang et al., 2025).

MC treatment blocks GA synthesis, thereby rebalancing the endogenous hormone system and affecting gene expression and organ development in cotton (Peng et al., 2025). Our previous studies showed that MC reduced plant height and internode length by suppressing GA biosynthesis and cell elongation in cotton (Wang et al., 2014). Transcriptome analysis of internodes revealed that MC not only downregulated genes related to GA but also significantly suppressed those involved in auxin, brassinosteroid, and ethylene metabolism and signaling, whereas genes associated with cytokinin (CK) and abscisic acid (ABA) metabolism were upregulated in response to MC (Wang et al., 2020, 2021a).

Ascorbic acid (AsA) functions not only as an antioxidant that scavenges reactive oxygen species and maintains redox homeostasis but also in plant growth and development, ion transport, phytohormone synthesis, signal transduction, and as a coenzyme in the hydroxylation of various compounds (Wang et al., 2021b). Horemans et al. (2000) report that AsA is closely related to GA because it serves as a coenzyme for GA 20-oxidase (GA20ox), a key enzyme in GA biosynthesis. The level of AsA is regulated by ascorbate oxidase (AO), which is responsible for the oxidative degradation of AsA (Singh et al., 2024). AO has been found to be mainly distributed in rapidly growing tissues and to be associated with many hormone-mediated processes (De Tullio et al., 2013; Chatzopoulou et al., 2020). AO plays important roles in plant growth and development (Mellidou and Kanellis, 2024). Silencing of *CmAO4* accelerates the ripening of melon fruits by enhancing ethylene production (Chatzopoulou et al., 2020). Garchery et al. (2013) revealed that a large number of auxin-responsive genes were affected in *AO* RNA-silencing tomato lines. Salicylic acid (SA) can inhibit the growth of tobacco plants by decreasing *AO* transcript levels (Wang et al., 2021b).

Foliar spraying is the primary method of MC application in plants, alongside seed soaking (Abbas et al., 2022). Following foliar absorption, MC is rapidly translocated throughout the plant to exert its physiological effects (Wang et al., 2014). As the first organ to perceive MC, leaves play a critical role in the response to MC (Peng et al., 2025). However, the molecular mechanisms underlying MC-induced changes in leaf traits, such as reduced leaf area, increased chlorophyll content, and enhanced photosynthetic rate, remain uncharacterized. In our previous work, we investigated the molecular mechanism by which MC inhibits cotton internode elongation at both the transcriptional and post-transcriptional levels, revealing a complex regulatory network (Wang et al., 2020; 2021a). The present study aims to uncover the regulatory mechanisms by which MC modulates cotton leaf growth and development through an integrated analysis of morphological, physiological and transcriptomic data, together with virus-induced gene silencing (VIGS). This study will advance our understanding of the molecular mode of MC and provide novel insights into its rational application in agricultural practice.

## 2 Materials and methods

### 2.1 Plant materials and treatments

Cotton (*G. hirsutum* cv. Zhongmian 49) seeds, provided by the Institute of Cotton Research, Chinese Academy of Agricultural Sciences (Anyang, China), were soaked in distilled water for 10 h at 27 °C and then sown in plastic pots (top diameter, 15 cm; bottom diameter, 14 cm; depth, 10 cm) filled with a growth substrate consisting of potting soil (Pindstrup Substrate no. 4, Pindstrup Horticulture Ltd., Shanghai, China) and vermiculite (1:1, v/v), at approximately 15% moisture content. The pots were placed in a growth chamber maintained at 28 °C/20 °C (day/night) with a 14 h photoperiod, 60% relative humidity, and a light intensity of 550 µmol m^-^² s^-^¹. The seedlings were irrigated with Hoagland nutrient solution.

When cotton seedlings reached the three-leaf stage, they were randomly assigned to two groups, each with three biological replicates, consisting of 10 seedlings. Based on our previous research (Wang et al., 2014), an 80 mg/L MC solution was applied as a foliar spray. The control group was sprayed with distilled water. The third leaves (from bottom to top) of both the MC-treated and control plants were collected at 2, 5, 8, 10, and 13 days after treatment for analysis.

### 2.2 Measurement of leaf area, chlorophyll content, photosynthetic rate and AsA content

The length and width of the third leaves were measured at 0, 2, 5, 8, 10, and 13 days after MC application. Leaf area was calculated using the length–width coefficient method (coefficient = 0.75) (Jiang et al., 2020). The net photosynthetic rate (Pn) of the third leaves was measured using an LI-6400 portable photosynthesis system at 2, 5, 8, 10, and 13 days after treatment.

Chlorophyll content was determined spectrophotometrically using 95% ethanol and calculated according to the Lichtenthaler formula (Lichtenthaler and Wellburn, 1983). AsA content was determined using a commercial assay kit (Solarbio, Beijing, China).

### 2.3 Transcriptome processing and analysis

At 1 and 8 days after MC treatment, the third true leaves were collected from both the MC-treated and control groups, with three biological replicates per treatment (each replicate consisting of three seedlings). Transcriptome sequencing and subsequent bioinformatic analyses were conducted by Metware Biotechnology Co., Ltd. (Wuhan, China).

Total RNA was extracted from the samples, and its integrity and potential DNA contamination were monitored via agarose gel electrophoresis. RNA purity, concentration, and integrity were further rigorously assessed using a NanoPhotometer spectrophotometer, a Qubit 2.0 Fluorometer, and an Agilent 2100 Bioanalyzer. Libraries were constructed using the NEBNext^®^ Ultra™ RNA Library Prep Kit according to the manufacturer’s instructions. After the libraries passed quality assessment, sequencing was performed on the Illumina HiSeq platform. To ensure high-quality data, raw reads in FASTQ format were first processed using fastp (version 0.19.3) to remove adapter sequences and low-quality reads, thereby obtaining clean reads for downstream analysis. The clean reads were mapped to the cotton reference genome using HISAT (version 2.1.0). Gene alignment was quantified by using featureCounts (version 1.6.2), and gene expression levels were normalized as fragments per kilobase of transcript per million mapped reads (FPKM). Differential expression analyses between the two groups were performed using DESeq2 (version 1.22.1), with differentially expressed genes (DEGs) which were screened using the criteria of |log_2_ (fold change)| ≥1 and padj value ≤0.05. Functional annotation and enrichment analyses of the identified DEGs were performed using the Kyoto Encyclopedia of Genes and Genomes (KEGG) database.

### 2.4 Quantitative reverse-transcription-PCR analysis

To validate the RNA-seq results, 11 DEGs were randomly selected for qRT-PCR analysis. The specific primers used for qRT-PCR are listed in **Supplementary Table 1**. Data were obtained from three biological replicates, using the same RNA samples as those used for the RNA-seq analysis. Total RNA was extracted from leaf samples and reverse-transcribed into cDNA. qRT-PCR was performed on a Roche LightCycler 96 Real-Time PCR System in a 15 μL reaction volume containing 1.5 μL cDNA template, 7.5 μL AceQ qPCR SYBR Green Master Mix, 1.0 μL each of forward and reverse primers (10 μM), and 5.0 μL of sterile water. The thermal cycling conditions were as follows: initial denaturation at 95 °C for 5 min, followed by 40 cycles of 10 s at 95 °C and 30 s at 60 °C. *GhUBQ7* served as the internal reference control. Relative expression levels were calculated using the 2^−ΔΔCt^ method.

### 2.5 Measurement of hormone content

Fresh leaf samples were harvested, immediately frozen in liquid nitrogen, ground into a powder (30 Hz, 1 min), and stored at −80 °C until analysis. A 50 mg aliquot of the plant sample was weighed into a 2 mL plastic microfuge tube, frozen in liquid nitrogen, and extracted with 1 mL of methanol/water/formic acid (15:4:1, v/v/v). Then, 10 μL of an internal standard mixture (100 ng/mL) was added to the extract for quantification. The mixture was vortexed for 10 min and centrifuged for 5 min (12,000 r/min, 4 °C). The supernatant was transferred to clean plastic microfuge tubes, evaporated to dryness, reconstituted in 100 μL of 80% methanol (v/v), and filtered through a 0.22 μm membrane filter for LC-MS/MS analysis. The sample extracts were analyzed using a UPLC–ESI–MS/MS system (UPLC, ExionLC™ AD, https://sciex.com.cn/; MS, Applied Biosystems 6500 Triple Quadrupole, https://sciex.com.cn/).

### 2.6 Virus-induced gene silencing assay and physiological index measurement

The gene-specific primers for *GhAO1A/D* (*GH_A12G0340* and *GH_D12G0372*, collectively referred to as *GhAO1*) were designed to amplify 323-bp target fragments using cotton leaf cDNA as the template (**Supplementary Table 1**). The tobacco rattle virus (TRV) vector (pTRV2) was digested with KpnI and EcoRI restriction enzymes. Target fragments were ligated into pTRV2 via enzyme-mediated recombination, followed by transformation into *Escherichia coli* for positive colony screening, PCR verification, and sequencing. Validated recombinant plasmids were then introduced into *Agrobacterium tumefaciens* strain GV3101. Following overnight cultivation at 28 °C in YEP medium supplemented with 50 μg/mL kanamycin, 25 μg/mL gentamicin, 10 mM MES, and 20 μM acetosyringone, the cells were pelleted by centrifugation at 6,000 rpm for 10 min at room temperature. The pellets were then resuspended in infiltration buffer containing 10 mM MgCl_2_, 10 mM MES, and 200 μM acetosyringone. The resulting cell suspensions were incubated at room temperature for at least 3 h. Agrobacterium strains harboring the pTRV derivative, including pTRV: *GhCLA1* (positive control; provided by Gao et al., 2011), pTRV:00, and pTRV: *GhAO1* (OD_600_ = 1.5), were mixed with pTRV-RNA1 at a 1:1 ratio. The mixtures of *Agrobacterium* strains were infiltrated into the cotyledons of 8-day-old cotton seedlings. Seedlings were kept in darkness for 24 h and then grown at 23 °C under a 14-h light/10-h dark photoperiod. To determine whether *GhAO1* was specifically silenced in the pTRV: *GhAO1* plants, the homologs of *GhAO1* were identified in the *G. hirsutum* genome according to the method described in **Supplementary Data Sheet 1**. When the true leaves of plants inoculated with pTRV : *GhCLA1* displayed an albino phenotype, leaf samples from VIGS plants were collected for RNA extraction and silencing efficiency analyses. The expression levels of *GhAO1* and its eight homologs were detected by qRT-PCR (**Supplementary Table 1**). At the four-leaf stage, phenotypic observations were recorded photographically, and plant height, leaf area, and internode length were measured. The third true leaves were collected for AsA content quantification. Three biological replicates were performed per treatment.

The pTRV : *GhAO1* and pTRV:00 plants at the four-leaf stage were treated with 80 mg/L MC as a foliar spraying. The control group was sprayed with distilled water. Ten days after treatment, the same parameters were measured using the methods described above.

### 2.7 Statistical analysis

Statistical analyses were performed using SPSS 20.0 (IBM Corp., Armonk, NY, USA). Independent-samples *t*-tests were conducted, and *p <*0.05 was considered statistically significant. Additionally, one-way analysis of variance (ANOVA) was used to assess the statistical differences, and mean comparisons were performed using Duncan’s multiple range test (*p <*0.05). Error bars in all figures represent the standard error of the mean.

## 3 Results

### 3.1 MC delays the leaf development of cotton seedlings

**Figure 1:**
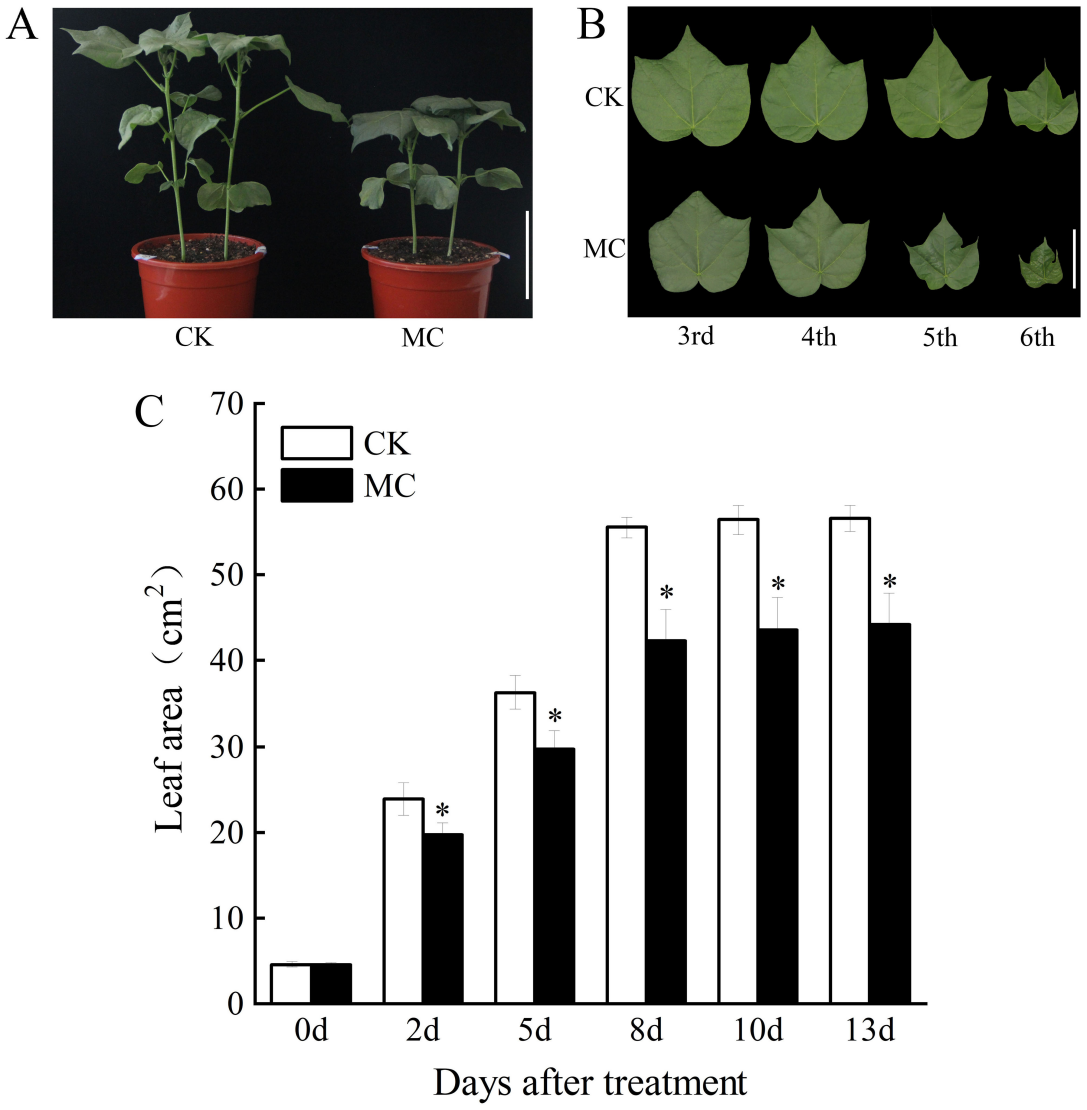
The effect of MC treatment on leaf expansion of cotton seedlings. **(A)** Phenotype of cotton seedlings at 10 days after MC treatment, scale bar = 10 cm. **(B)** Leaf morphology at 10 days after MC treatment, scale bar = 5 cm. **(C)** Dynamic changes of the third leaf (from bottom to top) area of cotton seedlings at different time points after MC treatment. Data are presented as mean ± SD (n = 3). *Indicates significant difference compared with the control (*p <*0.05). CK, untreated control; MC, 80 mg/L mepiquat chloride.

In this study, MC was applied when the third true leaf was fully expanded and the fourth true leaf remained unexpanded. Based on preliminary results and to ensure the integrity of the observations, the third true leaf was selected as the experimental sample. Plant height was significantly lower in the MC-treated group than in the control group (**Figure 1A**). MC treatment not only suppressed plant height but also significantly reduced leaf size compared with the control group (**Figure 1B**). In particular, the development of younger leaves (e.g., the sixth leaf) was more strongly inhibited by MC. For the third leaf, there was no difference in leaf area between the control and MC groups before treatment (**Figure 1C**). After MC treatment, leaf area was reduced by 17%–24% (*p <*0.05), and the difference in leaf area between the control and MC groups increased as the treatment duration extended until 8 days post-spray (dps), indicating 8 dps is a critical stage for leaf development. Combined with our previous experimental results (Wang et al., 2014; 2020; 2021a), 1 dps and 8 dps were selected as the sampling time points for transcriptome analysis.

### 3.2 MC enhances the chlorophyll content and net photosynthetic rate of cotton seedlings leaves

**Figure 2:**
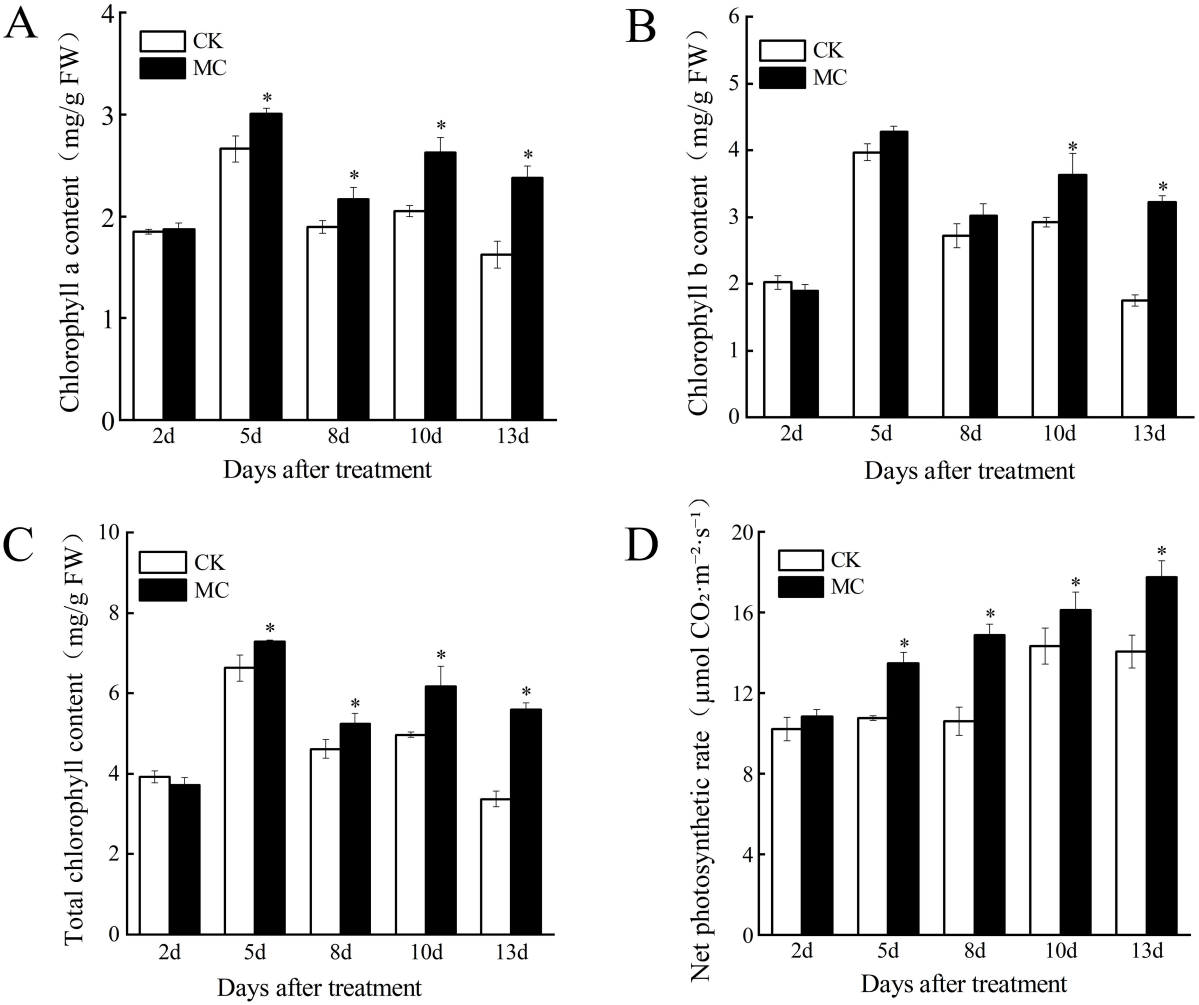
Effect of MC treatment on the chlorophyll content and the net photosynthetic rate of leaves in cotton seedlings. **(A)** Chlorophyll a content. **(B)** Chlorophyll b content. **(C)** Total chlorophyll content. **(D)** Net photosynthetic rate. Data are presented as mean ± SD (n = 3). *Indicates significant difference compared with the control (*p <*0.05). CK, untreated control; MC, 80 mg/L mepiquat chloride.

According to previous reports and the observations in this study (**Figure 1B**), cotton leaves exhibited a deep green color after MC treatment (Wu et al., 2019; Abbas et al., 2022). Therefore, chlorophyll content and net photosynthetic rate were examined. MC treatment significantly increased both chlorophyll content and the net photosynthetic rate (**Figure 2**). Chlorophyll content peaked at 5 dps in both groups. Chlorophyll a content increased by 13%–46% (*p <*0.05) from 5 days to 13 days after MC treatment (**Figure 2A**). Chlorophyll b content increased significantly from 10 dps onward (by 24%, *p <*0.05) and peaked at 13 dps (by 84%, *p <*0.05) (**Figure 2B**). Total chlorophyll content followed a trend similar to that of chlorophyll a (**Figure 2C**). The net photosynthetic rate increased gradually with leaf growth and was 25%, 40%, 13%, and 26% higher (*p <*0.05) in the MC group than in the control group at 5 dps, 8 dps, 10 dps, and 13 dps, respectively (**Figure 2D**).

### 3.3 Overview of the transcriptome profile of leaf in response to MC treatment

To investigate the molecular mechanism by which MC inhibits leaf development in cotton seedlings, RNA-seq was performed to profile the transcriptomic response of the third leaf to MC treatment. Twelve RNA libraries (leaf-CK, leaf-MC, at 1 day and 8 days after treatment) were constructed from the leaves of MC or water-treated seedlings and sequenced. After filtering, the valid read counts ranged from 41.97 to 55.13 million, the Q30 values ranged from 92.68% to 94.83%, and the GC content from 44.97% to 45.36% (**Supplementary Table 2**). Clean reads were aligned to the cotton genome, yielding unique mapping reads ranged from 89.12% to 91.61%.

### 3.4 Functional analysis of differentially expressed genes

**Figure 3:**
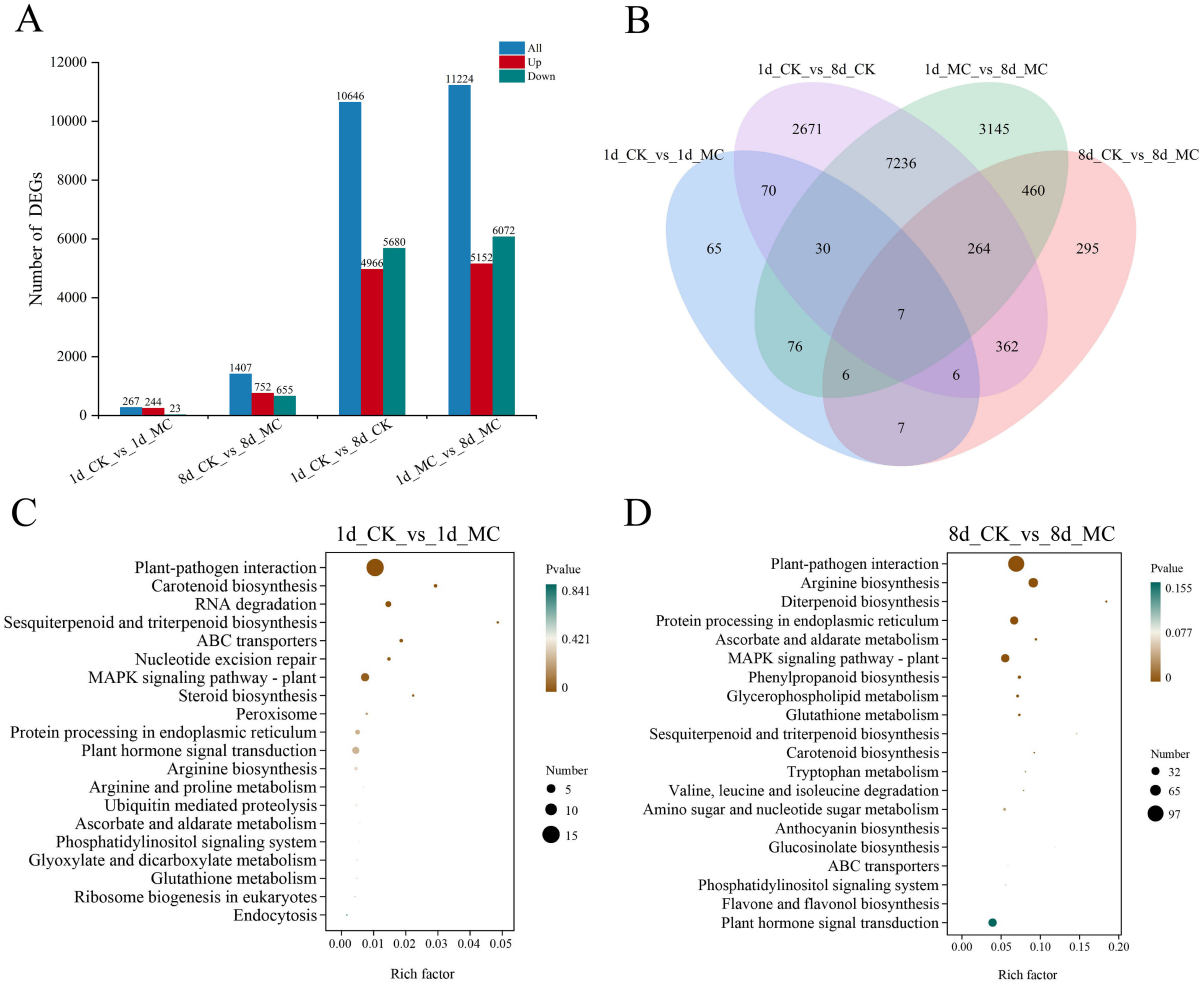
Number of DEGs in the leaf in response to MC treatment at different time points after treatment and KEGG pathway enrichment analysis of the DEGs. **(A)** The number of DEGs. **(B)** Venn diagram of DEGs. **(C)** Top 20 enriched KEGG pathways for DEGs identified in the 1d_CK vs 1d_MC comparison. **(D)** Top 20 enriched KEGG pathways for DEGs identified in the 8d_CK vs 8d_MC comparison. Y axis corresponds to the KEGG pathway, and the X axis shows the rich factor. The color of the dot represents the *p* value, and the size of the dot represents the number of DEGs mapped to the reference pathway.

To determine the changes in leaf gene expression resulting from MC treatment, gene expression was normalized using FPKM values. Transcripts with |log_2_(fold change)| ≥1 and a padj value ≤0.05 were considered as DEGs. Compared with the control group, 267 (244 upregulated and 23 downregulated) and 1,407 (752 upregulated and 655 downregulated) DEGs were identified in MC-treated leaves at 1 dps and 8 dps, respectively (**Figure 3A**). Venn diagrams showed that 26 DEGs were shared between the 1d-CK-vs-1d-MC and 8d-CK-vs-8d-MC comparisons, whereas 65 and 295 DEGs were uniquely identified in the 1d-CK-vs-1d-MC and 8d-CK-vs-8d-MC comparisons, respectively (**Figure 3B**). The results indicate that temporal changes primarily drove differential gene expression.

To better understand the potential functions of the DEGs in response to MC, KEGG enrichment analyses were performed for all pairwise comparisons. The top 20 KEGG pathways significantly enriched across all comparisons included plant–pathogen interaction, the mitogen-activated protein kinase (MAPK) signaling pathway–plant, plant hormone signal transduction, ascorbate and aldarate metabolism, carotenoid biosynthesis, and arginine biosynthesis (**Figures 3C, D**). Arginine and proline metabolism was uniquely enriched in the 1d-CK-vs-1d-MC comparison, whereas anthocyanin biosynthesis and flavone and flavonol biosynthesis were specifically enriched in the 8d-CK-vs-8d-MC comparison.

**Figure 4:**
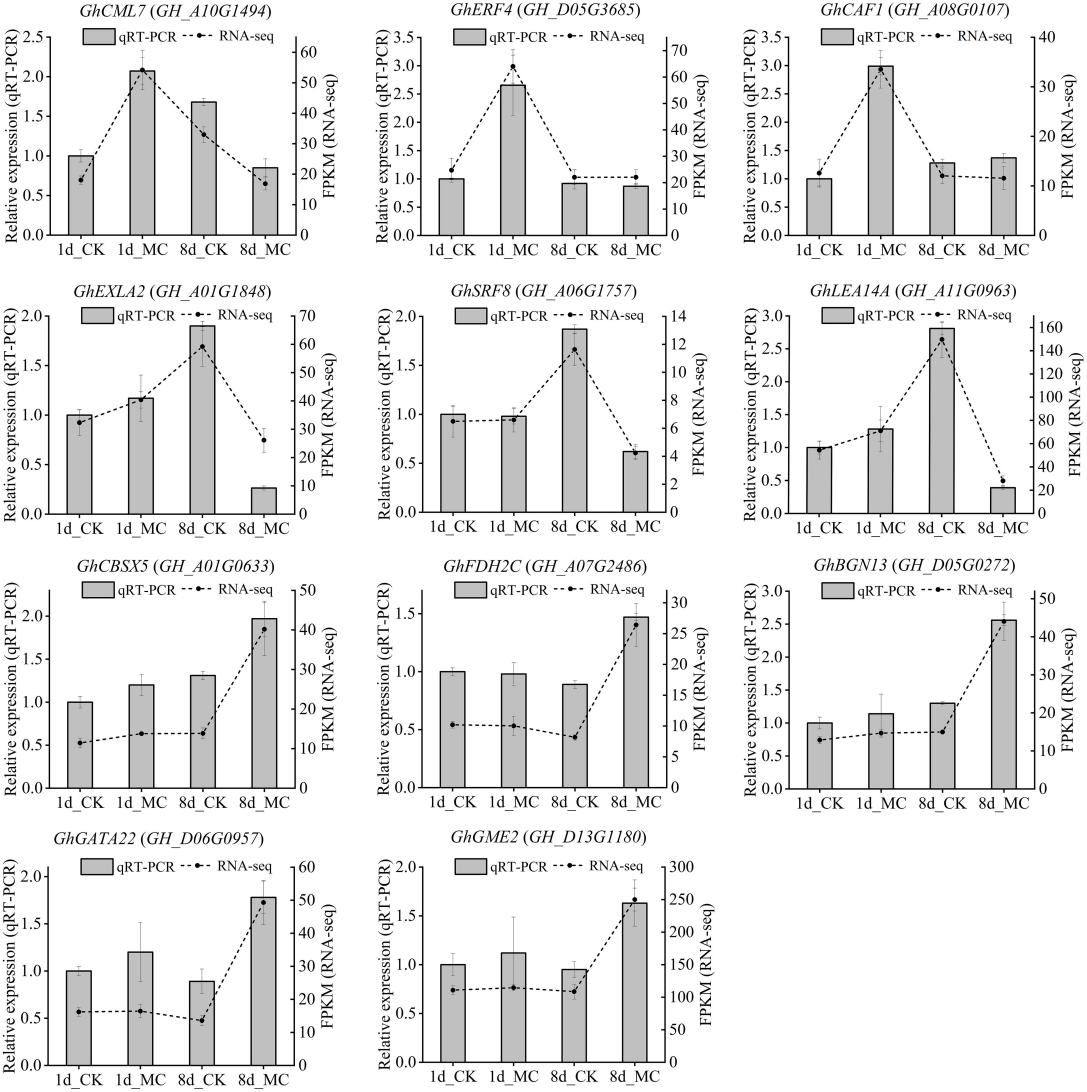
Validation of the expression levels of eleven DEGs by qRT-PCR. Comparison of RNA-seq data (dashed polyline) with qRT-PCR data (gray bar). The normalized expression level of RNA-seq (RPKM; reads per kilobase per million reads) is indicated on the right y-axis. The relative qRT-PCR expression level is shown on the left y-axis. Cotton *GhUBQ7* was used as a normalizer control. The mRNA level of each corresponding gene in 1d_CK was arbitrarily set to 1. CML7, calmodulin-like protein 7; ERF4, ethylene-responsive transcription factor 4-like; CAF1, CCR4-associated factor 1; EXLA2, expansin-like A2; SRF8, STRUBBELIG-RECEPTOR FAMILY 8-like isoform; LEA14A, late embryogenesis abundant protein Lea14-A; CBSX5, CBS domain-containing protein CBSX5-like; FDH2C, Formate dehydrogenase-2C mitochondrial; BGN13, glucan endo-1,3-beta-glucosidase 13-like; GATA22, GATA transcription factor 22; GME2, GDP-mannose 3,5-epimerase 2-like. Data are presented as mean ± SD (n = 3).

To validate the reliability of the RNA-seq, 11 DEGs were randomly selected for qRT-PCR analysis, including two genes showing differential expression only at 1 day after MC treatment, eight genes exhibiting differential expression exclusively at 8 days after MC treatment, and one gene displaying differential expression at both time points (**Figure 4**). Although the fold changes of the tested DEGs differed between RNA-seq and RT-qPCR, the up- or downregulation trends for the 11 tested DEGs were consistent with those in the RNA-seq data, confirming the reliability of the RNA-seq results.

### 3.5 DEGs related to hormone metabolism and signal transduction and changes in endogenous hormone levels

**Figure 5:**
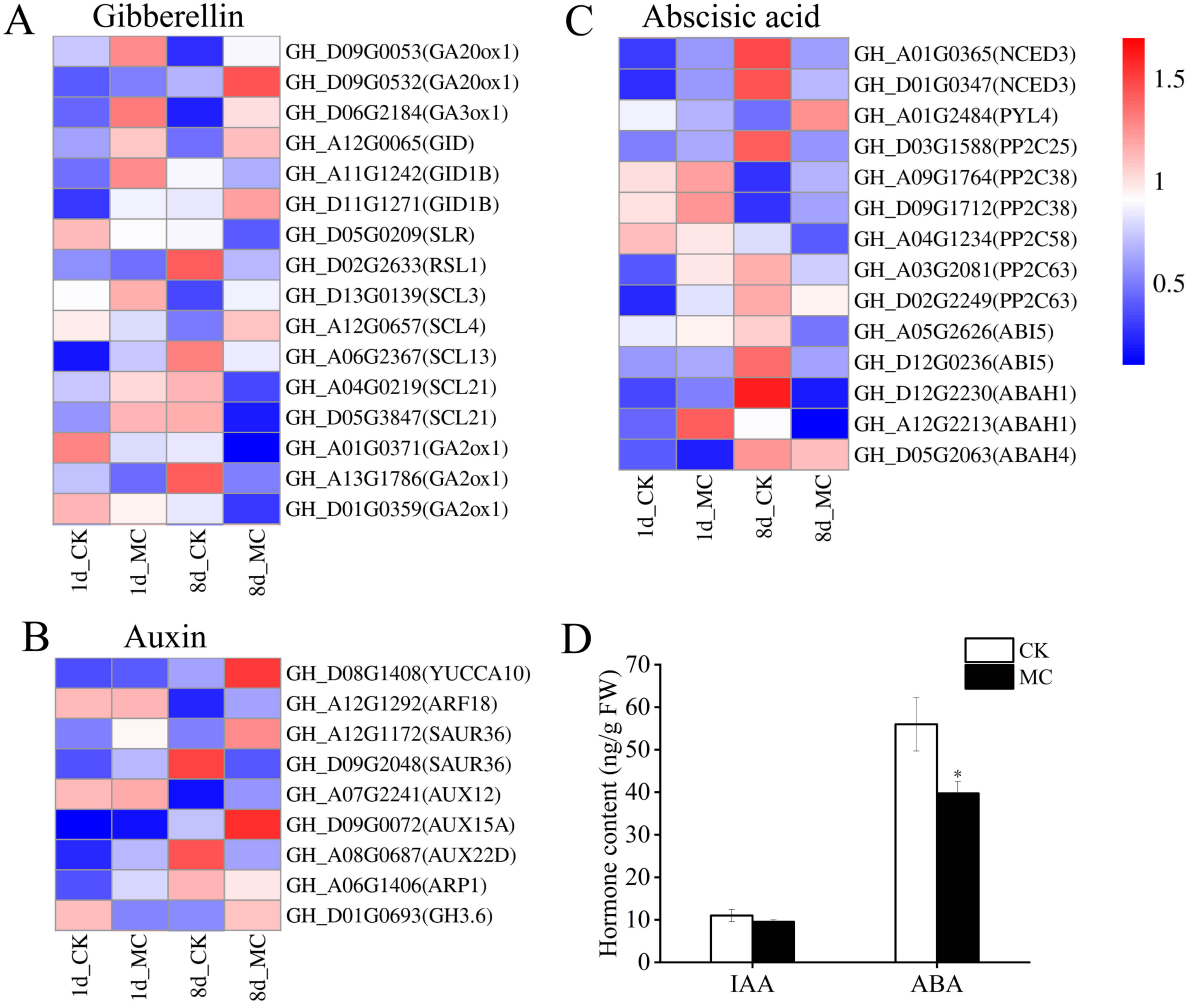
Heatmaps of DEGs involved in the metabolism and signaling of gibberellin **(A)**, auxin **(B)**, abscisic acid **(C)**, and hormone content **(D)**. Gene expression level (FPKM) is normalized. Blue indicates lower expression, and red indicates higher expression. The hormone content was measured in the third leaves of cotton seedlings at 8 days after MC treatment. Data are presented as mean ± SD (n = 3). ^*^Indicates significant difference compared with the control (*p <*0.05). CK, untreated control; MC, 80 mg/L mepiquat chloride.

To investigate the role of phytohormone in MC-mediated inhibition of leaf development, DEGs involved in the hormone metabolism, transport and signal transduction were identified by comparing the control and MC-treated groups. Thirty-nine hormones-related DEGs were detected after MC treatment, including GA-, ABA-, and auxin-related genes (**Figure 5**). Most of them exhibited decreased expression. Sixteen GA-related DEGs were found to be responsive to MC (**Figure 5A**). Three GA biosynthesis genes, including two *GA20ox* genes and one *gibberellin-3-β-dioxygenase* (*GA3ox*) gene, were upregulated, whereas three genes encoding GA-2-β-dioxygenase (GA2*ox*), which is involved in GA catabolism, were downregulated. Genes associated with GA signaling, such as *GIBBERELLIN-INSENSITIVE DWARF* (*GID*) genes and GRAS-family transcription factors (*SLR, SLENDER RICE*; *RSL1, RICESLEEPER 1-LIKE*; *SCL, SCARECROW-LIKE PROTEIN 3*), were predominantly downregulated. These findings indicate that MC suppresses GA signaling.

Auxin, a key regulator of leaf development, was also modulated by MC. The IAA biosynthesis gene, *indole-3-pyruvate monooxygenase* 10 (*YUCCA10*) and the IAA catabolism gene, *GRETCHEN HAGEN 3.6* (*GH3.6*) were both upregulated at 8 dps, whereas *GH3.6* was downregulated at 1 dps (**Figure 5B**). Genes involved in auxin signal transduction, including *AUXIN RESPONSE FACTOR18* (*ARF18*), *SMALL AUXIN-UP RNA 36* (*SAUR36*), AUXIN-INDUCED PROTEIN 12 (*AUX12*), *AUX15A*, *AUX22D*, and *AUXIN-RESPONSIVE PROTEIN 1*(*ARP1*), were predominantly upregulated.

Fourteen DEGs related to ABA metabolism and signaling were identified, including two ABA biosynthesis genes (*9-cis-epoxycarotenoid dioxygenase*, *NCED*), three ABA catabolism genes (*abscisic acid 8’-hydroxylase, ABAH*) and nine ABA signaling genes (*PYL*, *PYRABACTIN RESISTANCE-LIKE*; *PP2C*, *PROTEIN PHOSPHATASE 2C*; *ABI5*, *ABSCISIC ACID INSENSITIVE 5*) (**Figure 5C**). Among them, five genes were modulated by MC at 1 dps, most of which were upregulated. Eight genes were downregulated by MC at 8 dps, including two *NCED* genes, two *ABAH* genes, two *ABI5* genes, and two *PP2C* genes. These results indicate that ABA metabolism and signaling genes are primarily upregulated during the early stage and downregulated during the later stage following MC treatment.

To verify the regulation of endogenous hormones by MC, the contents of GA, indole-3-acetic acid (IAA), and ABA in the third leaves were measured 8 days after treatment. However, endogenous GA content could not be detected because of its relatively low abundance. This result suggests that MC effectively inhibited GA biosynthesis. IAA showed a slight decrease, whereas ABA was significantly reduced by 29% (*p <*0.05) (**Figure 5D**).

### 3.6 MC alters the expression of genes related to cell wall, AsA metabolism, and reduces AsA content

**Figure 6:**
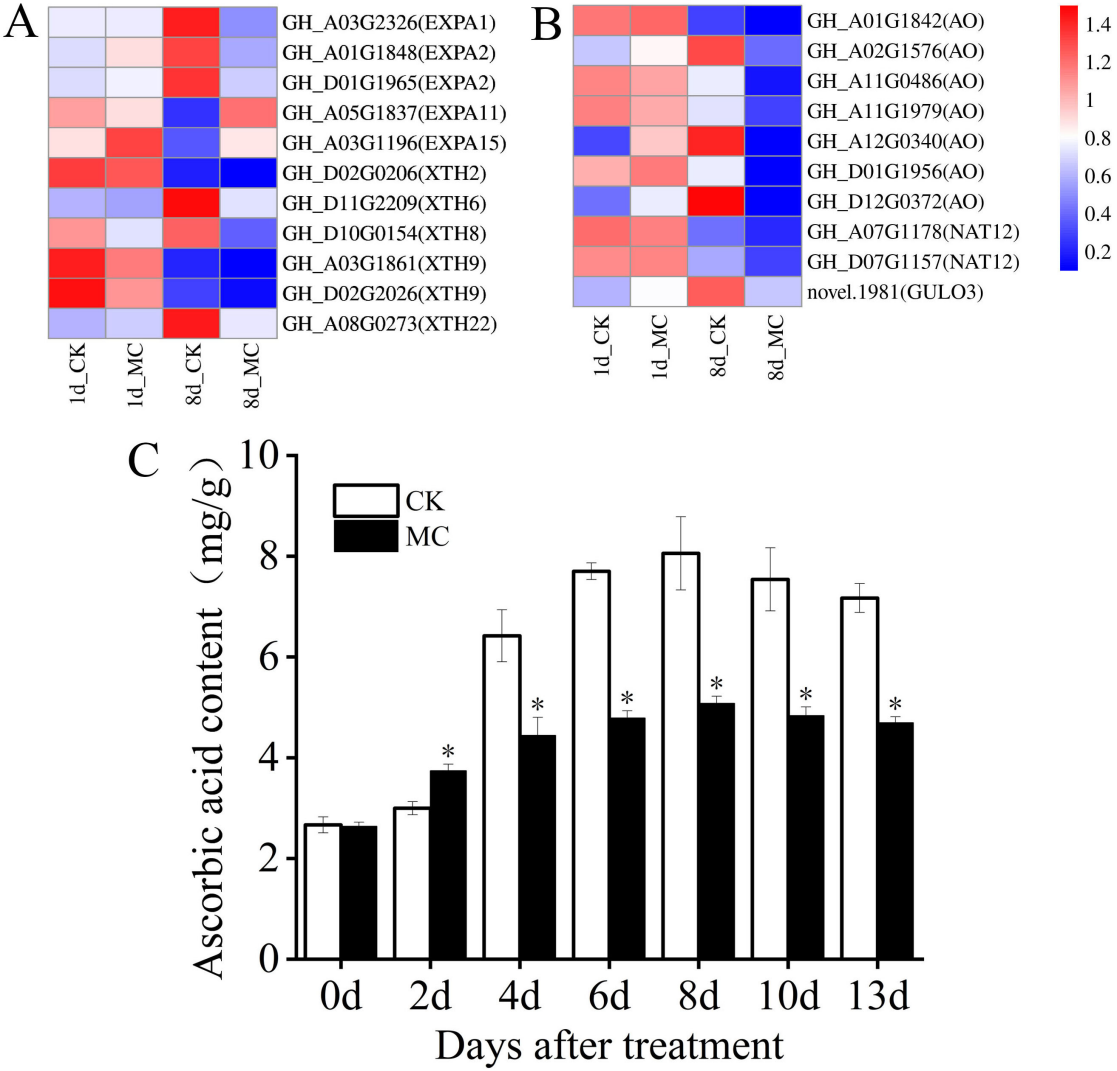
Heatmap showing the expression of DEGs related to cell wall expansion **(A)** and ascorbic acid metabolism and transport **(B)**, and dynamic changes of ascorbic acid content **(C)** in the leaf of cotton seedlings in response to MC. FPKM values are normalized. Blue indicates lower expression, and red indicates higher expression. Data are presented as mean ± SD (n = 3). *Indicates significant difference compared with the control (*p <*0.05). CK, untreated control; MC, 80 mg/L mepiquat chloride.

Consistent with the inhibition of leaf expansion, 11 DEGs encoding cell wall proteins were identified, including five *α-expansins* (*EXPA*s) and six *xyloglucan endotransglycolase/hydrolases* (*XTH*s) (**Figure 6A**). All of these genes were significantly downregulated by MC except *GhEXPA11* and *GhEXPA15*. KEGG analysis revealed that AsA metabolism was significantly enriched in response to MC treatment (**Figure 3**). To explore the role of AsA in MC-mediated growth regulation, AsA-related DEGs were identified. A total of ten DEGs were detected at 8 dps, including seven *GhAOs* and three genes related to AsA transport. All of *GhAOs* were significantly downregulated at 8 dps, whereas one *GhAO* (*GH_A12G0340*) was upregulated by MC at 1 dps (**Figure 6B**).

The altered expression of *GhAO* genes suggests that MC induces changes in leaf AsA content. To more accurately detect the dynamic changes in AsA content induced by MC application, the sampling interval was adjusted from every 3 days to every 2 days during the early stage of treatment, based on the other physiological measurements. During leaf development, AsA content gradually increased, peaked at 8 dps, and subsequently declined in both MC-treated and untreated leaves, suggesting that AsA is required for leaf development (**Figure 6C**). However, MC treatment slowed the rate of increase in AsA content from 2 dps to 8 dps. AsA content was reduced by 31%–38% from 4 dps to 13 dps (*p <*0.05), whereas it increased by 25% at 2 dps (*p <*0.05). The results indicate that MC may inhibit the leaf growth, at least in part, by disrupting AsA homeostasis.

### 3.7 Silencing of *GhAO1* prompts leaf expansion and reduces the sensitivity of plants to MC

**Figure 7:**
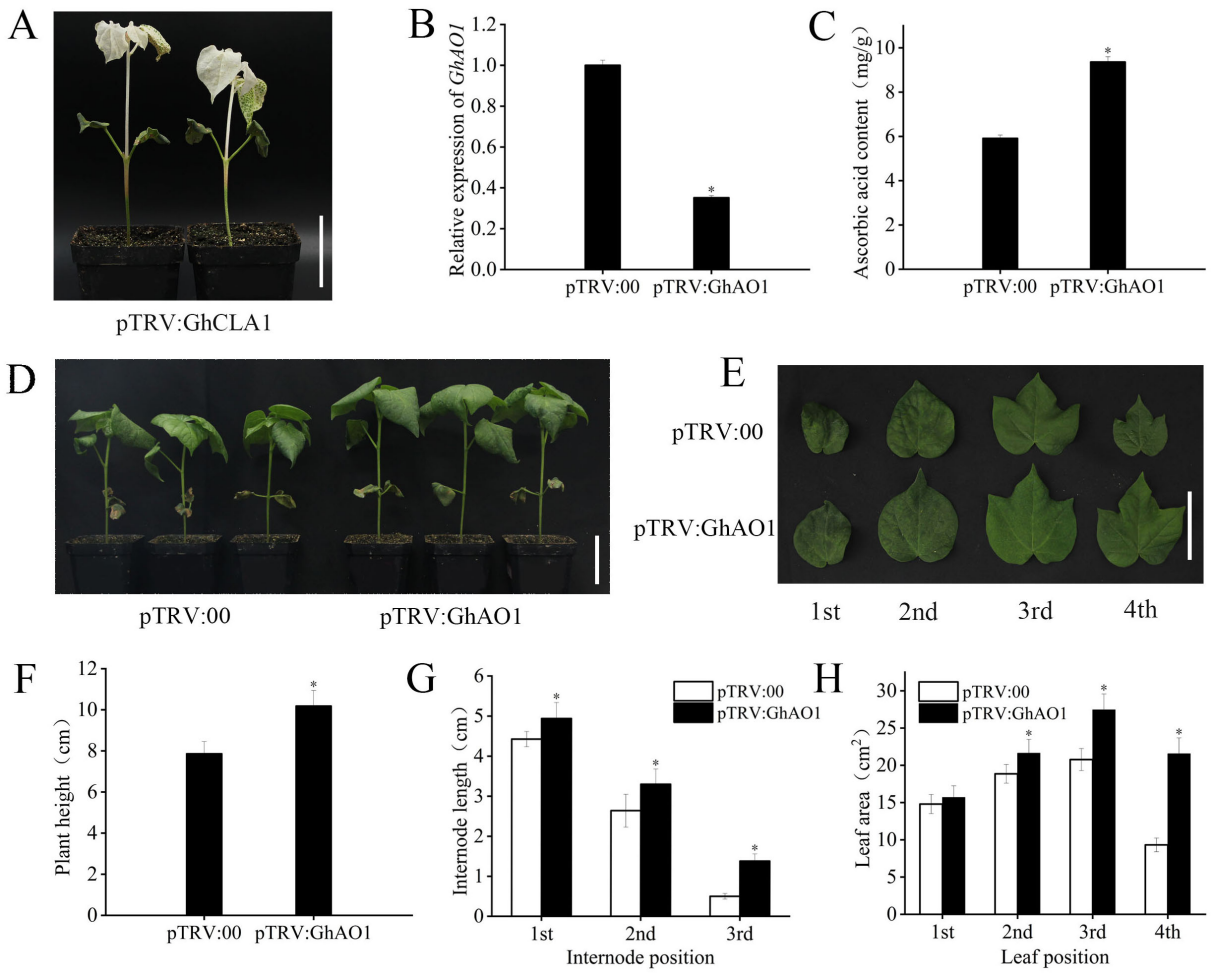
Silencing of *GhAO1* prompts leaf expansion and internode elongation. **(A)** The albino phenotype of pTRV: *GhCLA1* plants, scale bar = 5 cm. **(B)** Relative expression levels of *GhAO1* and **(C)** ascorbic acid content in pTRV:00 and pTRV: *GhAO1* plants. **(D)** Photograph of representative pTRV:00 and pTRV: *GhAO1* plants, scale bar = 5 cm. The 1st, 2nd, 3th, and 4th represent the position of leaf or internode from bottom to top in plant. **(E)** Leaf morphology of representative pTRV:00 and pTRV: *GhAO1* plants, scale bar = 5 cm. Plant height **(F)**, internode length **(G)**, leaf area **(H)** of pTRV:00 and pTRV: Gh*AO1* plants. *GhAO1* represents *GhAO1A/D*. The mRNA level of *GhAO1* in the pTRV:00 group was arbitrarily set to 1. Gene expression was normalized to the reference gene *GhUBQ7* and calculated using the 2^−ΔΔCt^ method. Data were presented as mean ± SD (n = 3). ^*^Indicates significant difference compared with the pTRV:00 group (*p <*0.05).

**Figure 8:**
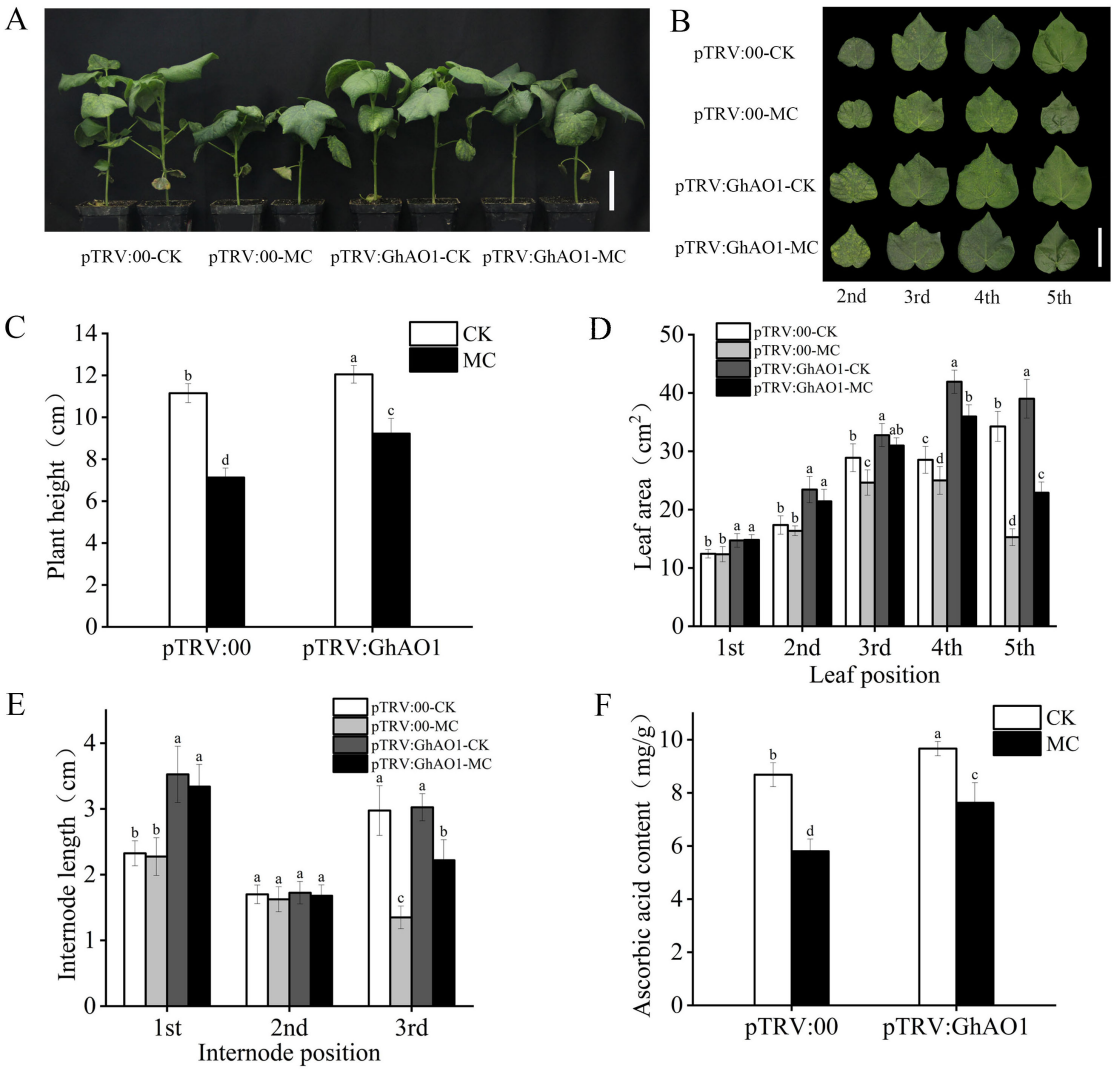
Silencing of *GhAO1* reduced the sensitivity of cotton seedlings to MC treatment. **(A)** Phenotypic appearance of pTRV:00 and pTRV: *GhAO1* plants at 10 days after MC treatment, scale bar = 5 cm. **(B)** Leaf morphology of pTRV:00 and pTRV: *GhAO1* plants at 10 days after MC treatment, scale bar = 5 cm. Plant height **(C)**, leaf area **(D)**, internode length **(E)**, and ascorbic acid content **(F)** of pTRV:00 and pTRV: *GhAO1* plants at 10 days after MC treatment. *GhAO1* represents *GhAO1A/D*. Data are presented as mean ± SD (n = 3). Different lowercase letters in the column denote the significance level of mean differences (*p <*0.05) among different groups. CK, untreated control; MC, 80 mg/L mepiquat chloride.

To further investigate the role of AO in leaf development, *GhAO1* (*GH_A12G0340* and *GH_D12G0372*) was selected for construction of the VIGS vector pTRV: *GhAO1*, which was subsequently introduced into cotton plants, with pTRV:00 as the control. The results showed that *GhAO1* expression was significantly suppressed (by 64.8%, *p <*0.05) in the corresponding VIGS cotton plants (**Figures 7A, B**), demonstrating high silencing efficiency. In addition, the expression of eight *GhAO* genes with high sequence homology to *GhAO1* was analyzed and showed no significant change in *GhAO1*-silenced plants (**Supplementary Figure 1**). *GhAO1-*silenced plants showed significant increases in AsA concentration, plant height, internode length and leaf area (*p <*0.05) (**Figures 7C–H**). Additionally, *GhAO1* silencing mitigated the inhibitory effects of MC on plant growth. Although MC treatment also reduced plant height, internode length, and leaf area in *GhAO1*-silenced seedlings compared with untreated plants, the reductions were smaller than those in pTRV:00 plants (*p <*0.05) (**Figure 8**). These findings not only demonstrate a strong association between plant development and AO-mediated AsA metabolism but also suggest that MC inhibits plant development, at least in part, by reducing AsA content.

## 4 Discussion

It is well established that MC treatment suppresses plant vegetative growth, resulting in reduced plant height and leaf area (Tung et al., 2020). Our previous work has shown that MC decreases plant height and internode length by inhibiting GA biosynthesis and cell elongation (Wang et al., 2014). Transcriptome analysis of cotton internodes revealed that MC downregulated multiple genes associated with cell growth and suppressed GA biosynthetic gene expression, thereby modulating the activity of other plant hormones and leading to shortened internode (Wang et al., 2020, 2021a). In the present study, we analyzed transcriptome data from the leaves of cotton seedlings and explored the molecular mechanism by which MC affects leaf development.

### 4.1 MC may serve as an abiotic stressor for leaves

In this study, leaves function not only as photosynthetic organs but also as primary sites for MC absorption and translocation. Our previous study indicated that after foliar application of MC, relatively high MC concentrations were maintained, with newly emerged leaves exhibiting higher MC levels than older leaves and internodes showing greater MC accumulation than unsprayed leaves and roots (Wang et al., 2014). At the beginning of MC application, leaves primarily absorb and translocate MC to other plant organs. During this stage, MC functions as a stressor, inducing shifts in metabolic pathways primarily involving ABC transporters, the isoflavone biosynthesis pathway, and plant hormone signal transduction (Wang et al., 2025). MC application increases the activity of antioxidant enzymes (superoxide dismutase, peroxidase) and the content of antioxidants (flavonoids) (Wang et al., 2025). Thus, MC acts not only as an inhibitor of GA activity but also as a potential abiotic stressor for leaves. Proline is a key osmoprotectant in plants under abiotic stress, particularly drought, suggesting that MC treatment induces localized stress in leaves, enhances MC translocation, alters osmotic potential, and consequently modulates proline-related gene expression and accumulation (Wang et al., 2024). The MAPK cascade is a highly conserved signaling pathway that senses diverse internal and external stimuli and regulates numerous biological processes. MAPK signaling plays a pivotal role in plant development and stress responses by linking upstream receptors to downstream effector proteins (Kumar et al., 2025; Yang et al., 2025). In this study, KEGG enrichment analysis revealed significant enrichment of the plant–pathogen interaction, ABC transporter, proline metabolism, and MAPK signaling pathways, further supporting the notion that MC may act as an abiotic stressor in leaves.

### 4.2 MC affects the metabolism and the signaling of other hormones by blocking GA biosynthesis

Our previous study demonstrated that treated leaves contained the highest MC levels following foliar application (Wang et al., 2014). *In vitro* experiments have shown that MC binds to an external site of the CPS active center, altering its molecular conformation and inhibiting CPS activity through noncompetitive inhibition, thereby reducing GA levels (Zhang et al., 2020). MC directly targets CPS to influence GA biosynthesis and also indirectly modulates GA biosynthesis by regulating genes involved in GA synthesis and degradation (Wang et al., 2022b). Bioactive GAs are maintained at stable levels through feedback regulation of GA metabolism in plants (Hedden and Phillips, 2000). Endogenous bioactive GA also regulate the expression of GA-related genes. Transcript levels of *GA20ox1*, *GA20ox2*, and *GA20ox3* are notably upregulated in *scl3* under GA-deficient conditions (Heo et al., 2011). GA catabolism genes are usually downregulated when GA levels decrease, whereas GA biosynthesis genes are upregulated (He et al., 2020). Elevated GA levels downregulate GA biosynthetic genes and upregulate GA catabolic genes (Fambrini and Pugliesi, 2013; Ritonga et al., 2023). Similar results were observed in this study (**Figure 5**). Although GA was not detected by LC-MC following MC treatment, MC promoted the expression of key GA biosynthetic genes (*GhGA20ox* and *GhGA3ox*), while suppressing that of key GA catabolic gene *GhGA2ox*. This finding indirectly suggests that MC reduces GA levels in cotton leaves. These findings are consistent with previous reports describing the feedback regulation of GA biosynthetic and catabolic genes by endogenous GA levels in plants (Heo et al., 2011). Transcriptome analysis indicates that the effects of MC on other plant hormones in leaves are consistent with our previous findings in cotton seedling internodes (Wang et al., 2021a). This further supports the conclusion that MC blocks GA biosynthesis in leaves, which in turn regulates the expression of GA metabolism genes through feedback mechanism to maintain endogenous hormone homeostasis.

### 4.3 MC retards cotton seedling development partly through disturbing AsA balance

AsA fulfills multiple roles in plants. As a substrate for AsA peroxidase, AsA reduces oxidants such as hydrogen peroxide, functioning as an antioxidant and contributing to the maintenance of redox homeostasis (Mellidou and Kanellis, 2024). Redox homeostasis is closely linked to the regulation of gene expression. Therefore, AsA participates in the regulation of gene transcription and protein translation (Horemans et al., 2000). As a cofactor for various enzymes involved in hormone biosynthesis and protein synthesis, AsA is implicated in plant hormone crosstalk, as well as in the synthesis, elongation, and cross-linking, thereby regulating plant growth (Singh et al., 2024). AsA serves as a cofactor for GA20ox, directly influencing its activity and thereby participating in GA biosynthesis (Lange, 1994). Cell division and expansion are critical processes during leaf development. GA regulates leaf size by modulating cell division and osmotic pressure (Fambrini and Pugliesi, 2013). AsA is predominantly localized in actively growing tissues and participates in cell division and expansion. Furthermore, as a cofactor, AsA promotes the hydroxylation of cell wall proteins, thereby facilitating their synthesis (Córdoba and González-Reyes, 1994). Expansins are key regulators of cell wall relaxation and remodeling, promoting cell wall loosening through a pH-dependent, non-enzymatic mechanism (Kong et al., 2019). During leaf development, expansin activity correlates with the relative leaf elongation rate (Feng et al., 2025). XTHs are enzymes involved in cell wall remodeling that participate in the construction and modification of cellulose–xyloglucan cross-links and serve as key mediators of plant cell dynamics during growth, development, and stress responses (Shen et al., 2025). Overexpression of the *BdXTH27* gene in *Brachypodium distachyon* increases cellulose levels and decreases hemicellulose levels (Feng et al., 2025), whereas downregulation of *PheXTH3* in *Phyllostachys edulis* inhibits cell wall thickening in vessel and fiber cells (Xie et al., 2025). Our previous study demonstrated that MC reduces the number of internode cells and inhibits cell growth (Wang et al., 2021a). The present study demonstrates that MC regulates cotton leaf growth by modulating AsA levels, and the expression of *AO*, *EXPA*, and *XTH* genes.

AO was first discovered in cabbage, and most studies on AO have also focused on vegetables and fruits (Chatzopoulou et al., 2020). Similar to AsA, AO is predominantly found in rapidly growing tissues but is localized to the cell wall (Wang et al., 2023). AsA is synthesized in the cytoplasm, and apoplastic AsA is primarily transported from the cytoplasm to the apoplast via membrane AsA transport (Mellidou and Kanellis, 2024). As a substrate of AO, AsA is oxidized by AO, which reduces O_2_ to H_2_O, thereby regulating the AsA pool size and influencing plant growth. In turn, AsA also affects AO activity. AO and AsA coordinately regulate the redox state of plants (Wang et al., 2023). *AO* transcription is regulated by plant developmental status and environmental stimuli, with species-specific differences reported (Singh et al., 2024). Among plant hormones, IAA, ethylene, and SA are the primary regulators of *AO* expression (De Tullio et al., 2013). Increased AsA levels under stress conditions leads to the upregulation of *AO* expression (Wang et al., 2023). Silencing *GhAO1* via VIGS significantly reduces pectin and lignin contents in cotton plants, thereby decreasing plant resistance (Kong et al., 2019). Correspondingly, *GhAO1* silencing altered the sensitivity of cotton seedlings to MC, indicating that AO is involved in MC-mediated regulation of plant growth (**Figure 8**). This also suggests that *AO* gene expression can, in turn, be regulated by AsA concentration to maintain AsA homeostasis (Mellidou and Kanellis, 2024). The experimental results indicate that MC treatment reduces AsA concentration, downregulates the expression of AsA transport genes and *AO* genes, and alters the redox status of cotton seedlings. Consequently, MC treatment inhibits the expression of *EXPA* and *XTH* genes, thereby regulating leaf development in cotton seedlings.

### 4.4 MC enhances amino acid and secondary metabolism to increase chlorophyll content

As the primary photosynthetic organ, leaves are rich in photosynthetic pigments. Chlorophyll captures light and converts it into chemical energy during photosynthesis (Wang et al., 2024). Chlorophyll synthesis begins with glutamyl-tRNA and proceeds through a series of enzymatic reactions to produce chlorophyll a, a portion of which is converted to chlorophyll b by chlorophyll a oxygenase (Ritonga et al., 2023). Chlorophyll content in leaves initially increases and subsequently declines during leaf development. MC application increases chlorophyll content without altering its developmental trend (Peng et al., 2025). Similar results were obtained in the present study. Chlorophyll synthesis requires amino acids and secondary metabolites, and both amino acid metabolism and secondary metabolism influence chlorophyll content (Lu et al., 2024). KEGG pathway analysis indicated that multiple amino acid metabolism and secondary metabolism pathways rank among the top 20 enriched pathways following MC treatment. Our previous study has shown that, in cotton internodes, MC treatment progressively increases the abundance of photosynthetic antenna proteins and enhances secondary metabolic pathways over time based on KEGG analysis (Wang et al., 2021a). Thus, MC may increase chlorophyll content by enhancing amino acid and secondary metabolism. MC-induced leaf area reduction may increase the concentration of chlorophyll biosynthetic enzymes per unit volume, thereby enhancing the rate of chlorophyll synthesis and increasing chlorophyll content. At the molecular level, MC treatment may increase chlorophyll content by enhancing amino acid and secondary metabolism.

## 5 Conclusion

MC, a widely used plant growth regulator, significantly improves plant growth and crop yield. However, its mechanism of action is complex and influenced by numerous factors. Different plants exhibit varying sensitivities to MC, and even within the same species, responses may differ. Elucidating its mechanism of action is crucial for the effective application of MC. In this study, transcriptome analysis and VIGS were employed to investigate the molecular mechanism by which MC affects leaf development. MC may act as an abiotic stressor during the initial stage after application, thereby altering stress-responsive metabolic pathways in leaves. By reducing GA levels, MC suppresses the expression of genes related to leaf development, thereby inhibiting leaf growth. AO is partially involved in MC-mediated regulation of leaf growth. MC may increase chlorophyll content by enhancing amino acid metabolism and secondary metabolism. These findings contribute to a better understanding of the mechanism of action of MC. However, this study finds that the MC-induced increase in leaf chlorophyll content was not accompanied by concomitant changes in the expression of chlorophyll metabolism-related genes, a finding that warrants further investigation.

## Data Availability

The datasets presented in this study can be found in online repositories. The names of the repository/repositories and accession number(s) can be found in the article/**Supplementary Material**.

## Supplementary material

Supplementary Table 1 Primers used for quantitative reverse-transcription-PCR and virus-induced gene silencing.

Supplementary Table 2 Summary of sequenced reads and mapping results of RNA-Seq libraries. CK represents control (treated with distilled water); MC represents mepiquat chloride treatment (80 mg/L); 1d and 8d represent samples collected at 1 and 8 days post-spraying, respectively.

Supplementary Figure 1 Phylogenetic relationships of the *AO* gene family and the expression of genes highly homologous to *GhAO1* in *Gossypium hirsutum*. **(A)** Phylogenetic tree of the *AO* gene family in *G. hirsutum*. The neighbor-joining (NJ) phylogeny tree was created using MEGA (version 12) (bootstrap value = 1000). *GhAO1* represents *GhAO1A/D* (*GH_A12G0340* and *GH_D12G0372*) that are shown in bold. **(B)** The expression levels of eight *GhAO* genes highly homologous to *GhAO1* in *GhAO1*-silenced cotton plants by qRT-PCR. The mRNA level of each corresponding gene in the pTRV:00 group was arbitrarily set to 1. Gene expression was normalized to the reference gene *GhUBQ7* and calculated using the 2^−ΔΔCt^ method.

Supplementary DATA SHEET 1 The methods for identification and evolutionary analysis of *AO* gene family members in *G. hirsutum*.
