# Supplementary material for: Involvement of ascorbate oxidase in the regulation of the leaf development mediated by mepiquat chloride in cotton seedlings
Source: Front Plant Sci. 2026 Jul 31;17:1899637. doi: 10.3389/fpls.2026.1899637 (PMC13473380; doi:10.3389/fpls.2026.1899637)
Supplement: Supplementary file 1 [file DataSheet1.docx]

Identification and evolutionary analysis of AO gene family members in *G. hirsutum*

To exclude potential functional compensation arising from the redundant expression of homologous AO paralogs, a genome-wide identification of the AO gene family in *G. hirsutum* was conducted. The *G. hirsutum* genome data were downloaded from the NCBI Genome database (https://www.ncbi.nlm.nih.gov/datasets/genome/). Amino acid sequences of *Arabidopsis thaliana* AO proteins, retrieved from the TAIR database (https://www.arabidopsis.org/), served as queries for a BLASTP search against the *G. hirsutum* proteome, with an E-value threshold of 1×10^−5^. Hidden Markov Model (HMM) profiles for the conserved domains (PF07732, PF00394, and PF07731) of the AO gene family were obtained from the Pfam database (http://pfam.xfam.org/) and used to identify candidate genes in the cotton protein database using HMMER (version 3.0). Candidate protein sequences were subsequently submitted to SMART (http://smart.embl.de/) and NCBI CD-Search (https://www.ncbi.nlm.nih.gov/cdd/) to confirm the presence of conserved domains. For phylogenetic analysis, the amino acid sequences of the identified *G. hirsutum* AO proteins were aligned using ClustalW in MEGA (version 12). A phylogenetic tree was then constructed using the neighbor-joining (NJ) method with 1000 bootstrap replicates.
