# Supplementary material for: Involvement of ascorbate oxidase in the regulation of the leaf development mediated by mepiquat chloride in cotton seedlings
Source: Front Plant Sci. 2026 Jul 31;17:1899637. doi: 10.3389/fpls.2026.1899637 (PMC13473380; doi:10.3389/fpls.2026.1899637)
Supplement: Supplementary file 2 [file Table1.docx]

Table S1. Primers used for quantitative reverse-transcription-PCR and virus-induced gene silencing.

| Gene id | Gene annotation | Forward primers (5'-3') | Reverse primers (5'-3') | | Application | |
| --- | --- | --- | --- | --- | --- | --- |
| GH_A01G0633 | *CBSX5* | TCAAAACCAAGCTCAGCAACAAG | | AGCAGGGACAGGTGAAAAGAG | | qRT-PCR |
| GH_D06G0957 | *GATA22* | GGAAAACGGTGCTGGAGTTGC | | AGGATTGCAGCATCTTCAACGTC | | qRT-PCR |
| GH_D05G0272 | *Glu13* | TGGAAAAGGAACCGGAAAAAGC | | TCCAAAACTGCATGCATCATCG | | qRT-PCR |
| GH_D13G1180 | *SlGME2* | AGGGTGTCCGTGGTCGTAATTC | | TGCTTGAGTTCCCACGACCTTG | | qRT-PCR |
| GH_A07G2486 | *FDH2D* | GTTGTGTGGAGGGAGCCTTG | | AGCCAATCCCTGCTGTGAGA | | qRT-PCR |
| GH_A11G0963 | *LEA14A* | AGGACTTCGTGGTGGATAAGGTG | | TGTAGGGGTTGGAGACAGAGAC | | qRT-PCR |
| GH_A01G1848 | *EXLA2* | ATCGGCCGAGACATTTTGAAAC | | TCCAGTTTGCAGACCCAACC | | qRT-PCR |
| GH_A06G1757 | *SRF8* | TCATACATCCCCGGCTTCTG | | TCTTGCACCGCTCACCTTTC | | qRT-PCR |
| GH_A10G1494 | *CML7* | TGAGGTCCGTTTTGGAATCGC | | ACCGTCACCGTCTACATCCAC | | qRT-PCR |
| GH_D05G3685 | *RF4* | CAGAGCAGCACCGTGGAGTC | | AAACACTCTCCGCCGCACTC | | qRT-PCR |
| GH_A08G0107 | *CAF1* | ACGTGGAGCGAGATCCTCAC | | TCCAGACGACATCATCAGCTC | | qRT-PCR |
| GH_D12G0372 | *AO1D* | CCCAGAATCCCAGTCCCTT | | CACCATTAAGCCCATGACCAT | | qRT-PCR  qRT-PCR |
| GH_A12G0340 | *AO1A* |  |  |  |  |  |
| GH_D01G1956 | *AO2* | CAACTACTGCCACTCTTCATTACA | | AACTGCGTATCTTTGTTTCCC | | qRT-PCR |
| GH_A01G1842 | *AO3* | TTTCAACCACTGCCACTCT | | CTGCGTATCTTTGTTTCCCTTT | | qRT-PCR |
| GH_A01G1057 | *AO4* | GCAAGTGGACCAAGGCCTAA | | ATGCTTCCTACACGAAAAATTCCT | | qRT-PCR |
| GH_D01G1096 | *AO5* | TTATTACATTGCGGTCTCCAC | | CGTAGTGATATGAACCCTGTG | | qRT-PCR |
| GH_D05G1852 | *AO6* | ATACAACCTCCGTGATGCCG | | AGTAGTGCGTTCTTGGGGATC | | qRT-PCR |
| GH_A05G1817 | *AO7* | CAGATCAACCTGCTCAAGACTATTAC | | CTGATAGAGCGGGCTTGGTTC | | qRT-PCR |
| GH_A05G2551 | *AO8* | TTGTTTTATGCCGCCGCTTC | | GAGTGAATATCAGGGCCTGGG | | qRT-PCR |
| GH_D05G2573 | *AO9* | TCACCACTACTGCGACCCTT | | TGCTTCCCATTAACTTGTCCC | | qRT-PCR |
| GH_D12G0372 | *AO1D* | ATGGTGCGTAAAGGATGGAGC | | GGTGGAATGGGGCATGTAGTT | | VIGS |
| GH_A12G0340 | *AO1A* |  |  |  |  | VIGS |

Table S2. Summary of sequenced reads and mapping results of RNA-Seq libraries.

| Sample | Total raw reads | Clean reads | Q30(%) | GC content(%) | Unique mapped |
| --- | --- | --- | --- | --- | --- |
| 1d_CK1 | 44767174 | 41968040 | 93.22 | 45.36 | 37643261(89.70%) |
| 1d_CK2 | 49564198 | 46851088 | 92.75 | 45.20 | 42419112(90.54%) |
| 1d_CK3 | 44463748 | 40489788 | 93.28 | 45.34 | 36085802(89.12%) |
| 1d_MC1 | 47687836 | 43906130 | 93.33 | 45.04 | 39846070(90.75%) |
| 1d_MC2 | 48392528 | 44741678 | 93.23 | 45.11 | 40444192(90.39%) |
| 1d_MC3 | 47622062 | 44246406 | 93.23 | 45.19 | 39975846(90.35%) |
| 8d_CK1 | 47110218 | 44549154 | 93.13 | 45.19 | 40141278(90.11%) |
| 8d_CK2 | 47639588 | 45063684 | 92.87 | 45.18 | 40683148(90.28%) |
| 8d_CK3 | 49132892 | 46792166 | 92.68 | 44.97 | 42865524(91.61%) |
| 8d_MC1 | 46967032 | 44249270 | 93.26 | 45.23 | 39489268(89.24%) |
| 8d_MC2 | 58321682 | 55126450 | 94.96 | 44.91 | 49994463(90.69%) |
| 8d_MC3 | 48932990 | 46068932 | 93.63 | 45.24 | 41124710(89.27%) |

CK represents control (treated with distilled water); MC represents mepiquat chloride treatment (80 mg/L); 1d and 8d represent samples collected at 1 day and 8 days post-spraying, respectively.
